# GERONTOLOGICAL EDUCATION: APPLIED PERSPECTIVES AND PEDAGOGICAL APPROACHES

**DOI:** 10.1093/geroni/igz038.237

**Published:** 2019-11-08

**Authors:** Kelly Niles-Yokum

**Affiliations:** University of La Verne, La Verne, California, United States

## Abstract

The art and science of gerontological pedagogy is a balance of multidisciplinary paradigms that provide students with a pathway to the depth and breadth of the field of gerontology. This session will explore a variety of issues, including possibilities and challenges within the context of pedagogical approaches to gerontology education both in and out of the classroom. Topics include applied and theoretical perspectives and the what, why, and how gerontological educators do what they do and how it can impact the learning environment not only for students but for the community at large.

